# Thyrotoxicosis Secondary to Gestational Trophoblastic Disease: A Rare Complication With Clinical Relevance

**DOI:** 10.7759/cureus.94487

**Published:** 2025-10-13

**Authors:** Luis Antonio Rodriguez Arrieta, Duvan Alejandro Grisales Cano, Ana Maria Londoño Fonseca, Jorge Fernando Quintero Arrieta

**Affiliations:** 1 Endocrinology, Diabetes and Metabolism, Clínica de Inmunología y Genética, Medellín, COL; 2 Internal Medicine, Universidad Cooperativa de Colombia, Medellin, COL; 3 Medicine, Unidad Central Del Valle del Cauca, Tuluá, COL; 4 Obstetrics and Gynecology, University of Cartagena, Cartagena, COL

**Keywords:** beta-hcg, gestational thyroid dysfunction, gestational trophoblastic diease, hyperthyroidism, thyrotoxicity

## Abstract

Thyrotoxicosis is a clinical syndrome defined by excessive exposure to circulating thyroid hormones, primarily triiodothyronine (T3) and thyroxine (T4). Its most common cause is hyperthyroidism, which is characterized by the increased production and secretion of hormones by the thyroid gland. However, it can also originate from extrathyroidal stimulation. An example is the disproportionately high production of human chorionic gonadotropin (HCG) in gestational trophoblastic disease (GTD), where excessive levels of this hormone induce thyrotoxicosis through cross-activation of thyroid-stimulating hormone (TSH) receptors. We present the case of a 23-year-old woman with a history of ectopic pregnancy who presented with persistent pregnancy symptoms. Clinical and laboratory studies confirmed GTD complicated by thyrotoxicosis, evidenced by markedly elevated levels of HCG, suppressed TSH, and elevated free T4. The imaging studies were consistent with an invasive mole with pulmonary metastasis. Management included antithyroid therapy with methimazole (thionamide), beta-blockers, and systemic chemotherapy, given the characteristics of the neoplasm.

This case highlights the importance of a multidisciplinary approach in rare conditions such as GTD associated with thyrotoxicosis, where endocrine stabilization and targeted chemotherapy are the fundamental pillars for optimizing clinical and oncological prognosis.

## Introduction

Gestational trophoblastic disease (GTD) is a rare complication of pregnancy characterized by abnormal proliferation of the trophoblastic epithelium. Its presentation ranges from benign forms such as complete hydatidiform mole to frankly malignant neoplasias, including invasive mole or choriocarcinoma [1,2]. Regarding its origin, GTD may manifest after a molar pregnancy in 50% of cases; it may also arise after a spontaneous abortion or ectopic pregnancy (25%), as well as after full-term or premature pregnancies (25%) [3].

This pathology is characterized by a marked increase in the hormone human chorionic gonadotropin (HCG), which directly results in inappropriate stimulation of thyroid-stimulating hormone receptors (TSHR) and subsequently the development of thyroid disease. This endocrine complication is based on the structural similarity between these hormones and the inherent ability of HCG to stimulate thyroid receptors. Thus, once GTD is successfully treated, HCG levels normalize and hyperthyroidism usually resolves [1,3]. The following is a clinical case demonstrating the relationship between gestational trophoblastic disease and the development of thyrotoxicosis.

## Case presentation

A 23-year-old female patient with a history of high blood pressure, obesity, and a recent ectopic pregnancy was treated surgically three months prior to admission (without histopathological study of the surgical specimen). She consulted with persistent symptoms suggestive of pregnancy, such as nausea, vomiting, and abdominal pain. She was reassessed by the gynecology department, where a transvaginal ultrasound reported GTD, for which she was referred to a tertiary-level hospital.

On admission to the institution, the patient was hemodynamically stable (heart rate 124 beats/min, respiratory rate 17 breaths/min, blood pressure 156/95 mmHg); no signs of active bleeding or systemic involvement were documented. Admission laboratories showed beta-human chorionic gonadotropin (BHCG) 651,215 mIU/ml, markedly elevated; additional thyroid-stimulating hormone (TSH) suppressed with elevated free thyroxine (FT4) (Table 1); thyroid ultrasound with diffuse increase in size and heterogeneous echogenicity, without solid nodules; and thyroid Doppler in the inferior thyroid artery with systolic velocity of 44 cm/sec. Based on these findings, a diagnosis of hyperthyroid thyrotoxicosis secondary to trophoblastic neoplasia was made. Management was initiated with methimazole 15 mg/day and propranolol 40 mg every 12 hours, with FT4 monitoring to titrate methimazole.

**Table 1:** Patient's laboratories during hospitalization. TSH (Thyroid Stimulating Hormone), T4L (Free Thyroxine), T3 (Triiodothyronine), AST (Aspartate Aminotransferase), ALT (Alanine Aminotransferase), BT (Total Bilirubin), Hb (Hemoglobin).

Laboratories	Day 1	Day 8	Day 9	Day 10	Day 11	Day 13	Day 17	Reference range
TSH	0.006		0.011	0.005	0.006	0.006	0.005	0.55- 4.78 uIU/mL
T4L	3.4		2.14	1.99	1.83	1.71	1.67	0.89-1.76 ng/dL
T3				1.61	1.63	1.82	1.68	0.60-1.81 ng/dL
AST	28	872.22	336.36	143.29	93.29	57.41	23.16	0.01 - 34.00 U/L
ALT	17	384.80	259.38	197.92	137.2		29.64	10.00 - 49.00 U/L
BT			1.01	1.12	0.99	0.92	0.70	0.30 - 1.20 mg/dL
Hb		8.24	7.78	7.80	7.3	6.84	7.71	12.00 - 16.00 g/dL

**Figure 1:**
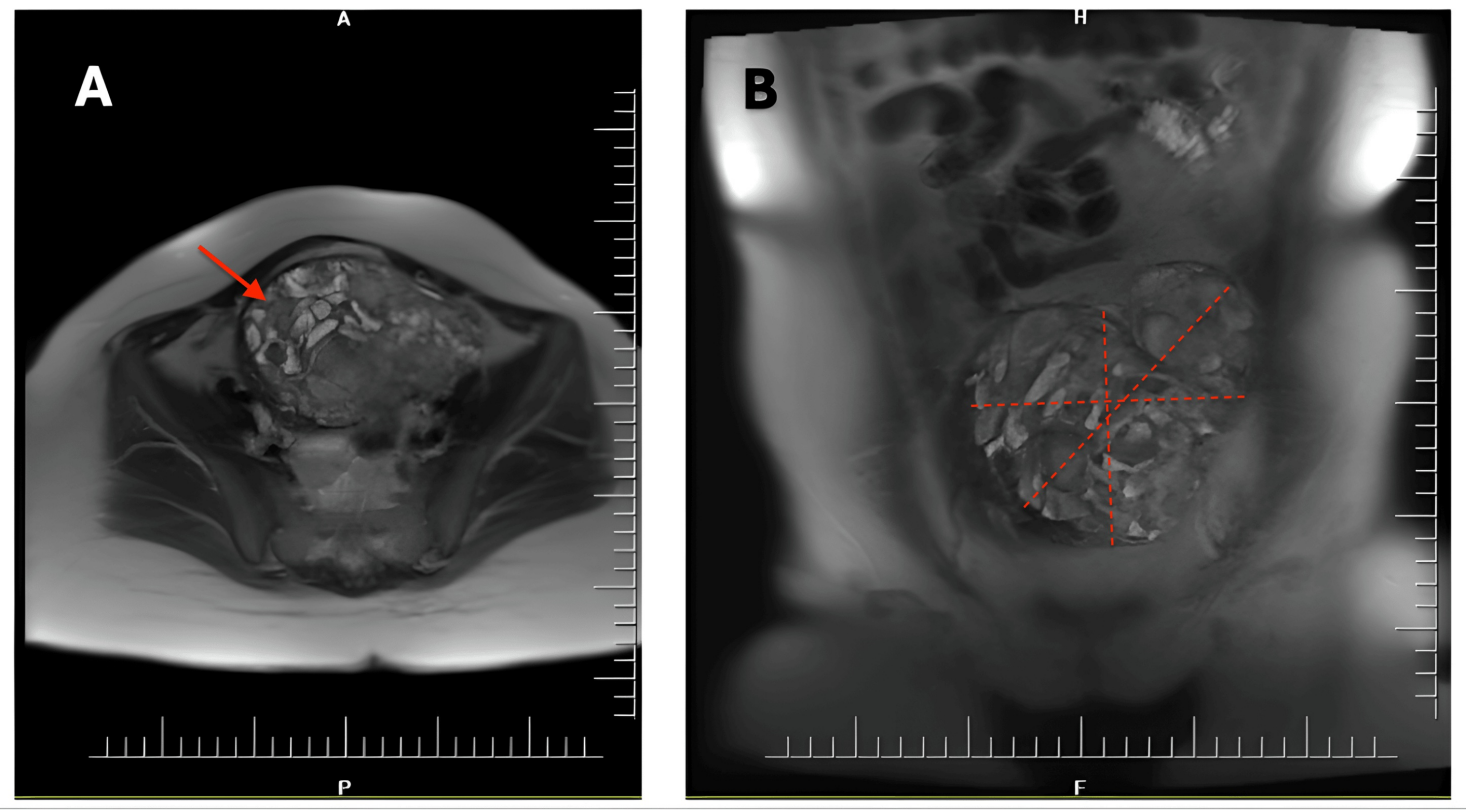
Contrast-enhanced magnetic resonance imaging of the abdomen and pelvis. A. Axial section showing a lesion with diffuse infiltration of the myometrium and endometrium, with multiple cystic-appearing images and vascular malformations. B. Coronal section showing marked restriction on diffusion-weighted sequences, measuring 14 cm in longitudinal diameter, 10 cm in anteroposterior diameter, and 12 cm in transverse diameter. Image provided from the patient’s medical records with prior authorization.

Contrast-enhanced magnetic resonance imaging of the abdomen and pelvis revealed a malignant tumor suggestive of an invasive mole (Figure 1).

No focal lesions suspicious for intra-abdominal metastasis or metastatic involvement in the central nervous system were identified; however, chest computed tomography documented solid nodular lesions suggestive of metastasis in the lung parenchyma.

The Oncology team, together with Gynecology, considered that, given the high vascularization of the mass and the lack of surgical viability for obtaining a biopsy, as well as the concordance between the tumor marker and the clinical and imaging findings compatible with gestational trophoblastic neoplasia, the initiation of systemic chemotherapy under the EMA/CO protocol (Etoposide, Methotrexate, Actinomycin D [EMA], and Cyclophosphamide and Oncovin [vincristine] [CO]) was the most appropriate strategy, with potentially curative intent. During hospitalization, she presented multiple complications, including a hemoperitoneum of 1,800 ml secondary to the rupture of the trophoblastic tumor, which required surgical interventions, embolization procedures, and transfusion support. After clinical stabilization, she was discharged from the hospital with an indication to continue outpatient follow-up by the oncology and endocrinology services.

## Discussion

Gestational trophoblastic disease (GTD) is a rare complication of pregnancy, the epidemiology of which is difficult to establish precisely due to its low incidence, the heterogeneity of tumor subtypes, and the limited availability of centralized databases [3]. The incidence of hydatidiform mole varies between 1 and 2 cases per 1,000 pregnancies in North America, Europe, and other developed countries, while in some Asian countries, as well as in Brazil and India, this is two to three times higher [4,5]. As for choriocarcinoma, its incidence is estimated at approximately three cases per 100,000 pregnancies in Europe and North America, in contrast to about 23 cases per 100,000 pregnancies in Southeast Asia [4,5]. These entities are characterized by their invasive and metastatic potential, with frequent involvement of the lung (80%), vagina (30%), brain (10%), and liver (10%) [6]; the most relevant complications are bleeding, trophoblastic pulmonary embolism, preeclampsia, or hyperthyroidism; the latter has been reported in 5% of molar pregnancies [7].

On the other hand, the overall prevalence of thyrotoxicosis ranges from 0.1% to 3.4% [8], with its most common causes being Graves' disease, toxic multinodular goiter, and toxic adenoma, while less common causes include thyroiditis, thyroid cancer, and the use of certain medications such as amiodarone [8]. However, the available literature does not report specific epidemiological data describing thyrotoxicosis and TGE.

In 1971, Hershman and Higgins made the first description of severe hyperthyroidism associated with hydatidiform mole when they documented two clinical cases in patients who presented thyrotoxicosis and pulmonary edema in the context of gestational trophoblastic disease. Both cases experienced rapid improvement after removal of the hydatidiform mole; additionally, the authors identified elevated levels of a thyroid stimulator, which they named at that time chorionic thyrotropin [9]. Subsequent investigations demonstrated that thyrotoxicosis associated with gestational trophoblastic disease is explained by hormonal cross-reactivity, based on molecular structural homology. The underlying pathophysiological mechanism involves the ability of HCG to directly stimulate TSHR, generating an abnormal thyrotropic response [10]. 

**Figure 2:**
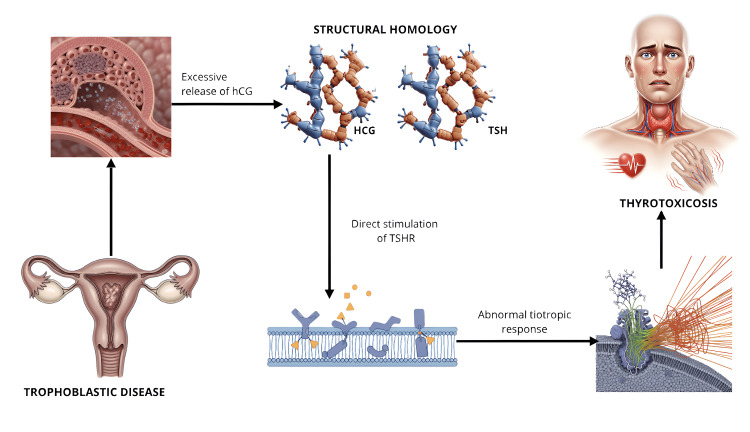
Pathophysiology of thyrotoxicosis associated with gestational trophoblastic disease. Note: Image created by the author using Gemini AI based on original instructions (copyright-free image). Final composition and numbering were performed in Canva. © 2025, Author’s own creation.

The molecular basis for this cross-reactivity is based on the significant structural homology between HCG and TSH, particularly in their alpha subunits. Both hormones belong to the family of heterodimeric glycoprotein hormones composed of noncovalently linked alpha and beta subunits. The alpha subunit of HCG is virtually identical to that of TSH, while the beta subunits, although similar, retain sufficient differences to confer biological specificity (Figure 2) [10,11].

Clinical manifestations of thyrotoxicosis include fever, diaphoresis, anxiety, insomnia, delirium, psychosis, stupor, coma, weight loss, and diarrhea. In the context of GTD, one of the most reported symptoms is multiple emetic episodes, especially in the presence of elevated HCG levels, a phenomenon also described in multiple pregnancies [12]. In the presented case, the patient experienced recurrent episodes of emesis, accompanied by hypertension and tachycardia. During hospitalization, she developed hemoperitoneum requiring surgical intervention and embolization. This complication is explained by the nature of the invasive mole, which is highly angiogenic and has a remarkable capacity to erode maternal blood vessel walls [10]. The abnormal trophoblastic proliferation deeply penetrates the myometrium, reaching medium- and large-caliber vessels, causing vascular ruptures and massive hemorrhage. Additionally, the generated vascular network is fragile and thin-walled, making it susceptible to rupture even without obvious trauma [10,13].

The main features associated with an increased risk of thyrotoxicosis include a fundal height greater than 16 cm, the presence of theca-lutein cysts greater than 6 cm, and serum HCG concentrations greater than 400,000 IU/L [14]. For choriocarcinoma, management is based on the International Federation of Gynecology and Obstetrics (FIGO) staging classification, which states: stage I, tumor confined to the uterus; stage II, extrauterine extension limited to the adnexa, vagina, and/or broad ligament; stage III, pulmonary involvement; and stage IV, involvement of other organs. The prognostic scoring system also defines low risk as a score of ≤6 and high risk as a score of ≥7 [3,14].

The optimal approach requires a multidisciplinary team that includes gynecology, oncology, endocrinology, and intensive care. In cases requiring radical surgical treatment by excision of the trophoblastic lesion, it is essential to identify and correct thyrotoxicosis before surgery, since untreated hyperthyroidism can precipitate acute clinical deterioration with high morbidity and mortality [14].

Symptomatic and antithyroid management constitutes a multimodal therapeutic approach. Therefore, thyroid hormone synthesis inhibitors, such as methimazole or propylthiouracil, are used to regulate excessive thyroid hormone production. In addition, peripheral conversion of T4 to the more potent hormone T3 can be inhibited by corticosteroid medications, with hydrocortisone and dexamethasone being the most frequently used agents. Along these lines, β-adrenergic blockers, particularly propranolol, can be used to reverse metabolic and cardiovascular characteristics [6]. Additionally, management of thyroid storm may include targeted fluid resuscitation, correction of electrolyte disturbances, oxygen therapy, iodine supplementation, and, in selected cases, plasmapheresis [6].

In the presence of active bleeding and marked hypervascularization, the surgical risk is considerably high. Therefore, in the absence of a vital indication, initial management is usually chemotherapy, since this not only controls tumor proliferation but also reduces vascularization, which decreases the risk of new bleeding and facilitates a possible surgical approach in refractory cases. The reported cure rates with this approach range from 86% to 100%, depending on the tumor stage and the prognostic score [3]. In such scenarios, concomitant management of hyperthyroidism is considered essential, as occurred in the case presented, in which the patient had thyrotoxicosis, opting for the administration of chemotherapy in conjunction with methimazole and propranolol, adjusting the therapy according to the clinical and biochemical response.

## Conclusions

Thyrotoxicosis secondary to gestational trophoblastic disease is a rare but clinically relevant complication, the pathophysiology of which is explained by the cross-activation of the TSH receptor induced by excessively high levels of hCG. The clinical manifestation of thyrotoxicosis in the context of gestational trophoblastic disease requires hCG levels above 100,000 IU/L. The use of methimazole and beta-blockers has proven effective in controlling thyrotoxicosis and providing symptomatic relief. These drugs can be discontinued once thyroid function has normalized after controlling the underlying disease. The case presented demonstrates how the early identification of this endocrine disorder, its appropriate pharmacological management, and systemic chemotherapy constitute the therapeutic cornerstone in highly vascularized trophoblastic neoplasia. It also highlights the importance of a multidisciplinary approach in complex scenarios, where the interaction between gynecology, oncology, and endocrinology is essential to reduce the risk of serious complications, improve metabolic control, and optimize cancer prognosis.
